# Interactions between the breast cancer-associated MUC1 mucins and C-type lectin characterized by optical tweezers

**DOI:** 10.1371/journal.pone.0175323

**Published:** 2017-04-17

**Authors:** Soosan Hadjialirezaei, Gianfranco Picco, Richard Beatson, Joy Burchell, Bjørn Torger Stokke, Marit Sletmoen

**Affiliations:** 1 Biophysics and Medical Technology, Department of Physics, NTNU Norwegian University of Science and Technology, Trondheim, Norway; 2 Breast Cancer Biology, King’s College London, Guy’s Hospital, London, United Kingdom; 3 Department of Biotechnology, NTNU Norwegian University of Science and Technology, Trondheim, Norway; Semmelweis Egyetem, HUNGARY

## Abstract

Carbohydrate–protein interactions govern many crucial processes in biological systems including cell recognition events. We have used the sensitive force probe optical tweezers to quantify the interactions occurring between MGL lectins and MUC1 carrying the cancer-associated glycan antigens mucins Tn and STn. Unbinding forces of 7.6±1.1 pN and 7.1±1.1 pN were determined for the MUC1(Tn)—MGL and MUC1(STn)—MGL interactions, at a force loading rate of ~40 pN/s. The interaction strength increased with increasing force loading rate, to 27.1±4.4 and 36.9±3.6 pN at a force loading rate of ~ 310 pN/s. No interactions were detected between MGL and MUC1(ST), a glycoform of MUC1 also expressed by breast carcinoma cells. Interestingly, this glycan (ST) can be found on proteins expressed by normal cells, although in this case not on MUC1. Additionally, GalNAc decorated polyethylene glycol displayed similar rupture forces as observed for MUC1(Tn) and MUC1(STn) when forced to unbind from MGL, indicating that GalNAc is an essential group in these interactions. Since the STn glycan decoration is more frequently found on the surface of carcinomas than the Tn glycan, the binding of MUC1 carrying STn to MGL may be more physiologically relevant and may be in part responsible for some of the characteristics of STn expressing tumours.

## Introduction

Glycosylated proteins and other glycoconjugates are major components of cells, defining and modulating several key physiological processes in normal tissues. Many of the effects of the glycoconjugates are mediated by glycan–lectin interactions, that are involved in involved in many normal and pathological processes from cell recognition and communication to pathogen invasion and tumour metastasis [1, 2]. The awareness of the glycan component of glycoconjugates carries biological information has motivated numerous studies of glycans, and significant progress has been made in the past years related to defining the structures and functions of glycans in biological systems. However, the progress within this field is challenged by the complexity and structural variation found in glycoconjugates combined with the high specificity, low affinity, and often multivalent nature of the interactions. There is therefore a need for new experimental techniques to study glycan related biological and medical problems [3]. Optical tweezers (OT) is one of several single-molecule manipulation techniques that have evolved rapidly over the last decades and that are finding an increasing number of applications within life-sciences. This technique is based on the generation of an optical trap, through focusing a laser to a diffraction-limited spot with a high numerical aperture microscope objective [4]. A dielectric particle near the focus will experience a restoring force that keeps the particle near the focus, as further outlined in several reviews [5, 6]. For small displacements of the particle, the optical trap acts as a linear spring. The calibration underlying the conversion from the detected displacement of the particle to the force driving this displacement is straightforward for silica and polystyrene beads, and these are therefore widely used in experiments aiming at determining interaction forces, as handles for the biomolecules of interest. OT have been applied for high resolution studies of forces required to unbind biomolecules [7], studies of structural dynamics of biomacromolecules [8] studies of individual molecular motors [9], as well as studies of mechanical properties of biological tissues and cells [10]. Despite these well documented capabilities of the OT, atomic force microscopy (AFM), characterized by a force range going from 5 to 1000 pN [5], is more frequently used to determine molecular interaction forces. However, the low strength of carbohydrate–protein and carbohydrate–carbohydrate interactions makes OT, capable of determining forces in the range going from 0.5 to 100 pN [5], an ideal probe for quantification of these interactions. The aim of the present paper is to characterize the unbinding properties of mucins carrying the cancer antigens ST and STn with MGL, including also identifying the carbohydrate moieties mechanistic in these interactions.

Mucins are glycoproteins that contain a range of *N-*acetylgalactosamine (GalNAc)-Ser/Thr *O*-linked glycans, and these glycans comprise more than 50 wt% of the molecule. Cell-surface-bound and secreted mucins from epithelial and other mucin-producing cells constitute an important part of the glycome surrounding these cells. Whereas secreted mucins function as a protective layer over the epithelium, the glycans of cell-surface-bound mucins control antigenicity as well as interactions with the environment and bind to mammalian lectins [11]. In this study we focus on the transmembrane, human mucin MUC1 [12]. MUC1 contains a variable number of tandem repeats (TRs) (25–125) of 20 amino acid residues with each repeat having five potential sites for O-glycosylation [13].

Certain changes in glycosylation are associated with development of cancer [14]. Cancer cells often express truncated glycan structures including the carbohydrate antigens Tn (GalNAcα1-*O*-Ser/Thr), the sialylated Tn structure (STn; NeuAcα2-6GalNAcα1-*O*-Ser/Thr), and T (Galβ1- 3GalNAcα1-*O*-Ser/Thr) [15]. The Tn structure has been described as a tumor-associated antigen in various human tumor entities [16]. It is regarded as a useful biomarker because it is expressed early in transformed cells, both in human [17, 18] and in animal carcinogenesis [19]. Furthermore, a direct correlation has been shown between carcinoma aggressiveness and the density of this antigen [20]. The presence of STn in human tumors can be due to the up-regulation of ST6GalNAc-I transferase [21, 22] or the inactivation of the COSMC chaperone [23]. In addition to these short cancer-associated antigens, MUC1 expressed by breast carcinoma cells also carries the mono- and disialyl core 1 structure (ST, NeuAcα2-3Galβ1–3[NeuAcα2–6]+/–GalNAcα1-*O*-Ser/Thr) found widely in normal cells [24–26]. Cancer-associated aberrant glycosylation can represent altered capacities for interaction with the microenviroment.

The interaction between tumor-associated antigens and specialized antigen-presenting cells is critical for the induction of a specific anti-tumour immune response. Glycopeptides corresponding to three tandem repeats of MUC1, glycosylated with 9 or 15 molecules of GalNAc, have been shown to specifically bind to and be internalized by immature monocyte-derived dendritic cells (DCs) [27]. The macrophage galactose-type lectin (MGL) expressed by monocytes is a well-studied C-type lectin binding to MUC1 [27]. Human MGL is a 40 kDa transmembrane glycoprotein consisting of a 39 amino acid (aa) cytoplasmic region, a 21 aa transmembrane segment and a 256 aa extracellular domain (ECD) with a carbohydrate recognition domain (CRD) and a neck region [28]. It is reported that MGL binds to the Tn antigen present on MUC1 [29], and NMR data indicate that MGL also binds the STn antigen [30]. Furthermore, based on NMR data it has been suggested that the affinity of the STn antigen to MGL is mainly mediated by the GalNAc moiety [30].

The presence of tumor-associated macrophages (TAMs) in the microenvironment of malignant tumors of human carcinomas has been correlated with an adverse prognosis of the patients [31]. A subpopulation of TAMs, the M2 macrophages, appear to be causally involved in the tumor progression [31, 32], and monocytes can be differentiated into M2 macrophages by addition of the conditioned tumor cell medium. Interestingly, monocytes stimulated in this way express MGL [33]. The interaction of MGL, expressed by M2 macrophages, with Tn and STn exposed by tumor cells, has been suggested to modulate the TAM phenotype and/or activity, and thus affect the progression of human tumors [30]. The importance of gaining further knowledge of the possible role of the STn structure is supported by the fact that the Tn glycan is mostly intracellular and not frequently on the carcinoma surface [34]. Thus, the binding of MUC1 carrying STn to MGL may be more physiologically relevant than the binding to MUC1 carrying Tn [35]. Tumour associated STn is associated with poor prognosis and resistance to chemotherapy in breast carcinomas [36], inhibition of DC maturation [37], DC apoptosis [38] and inhibition of NK activity [39], and the binding of MUC1(STn) to MGL may be in part responsible for some of the characteristics of STn expressing tumours.

In this paper we quantify and compare the strength of the molecular interaction between the two cancer associated antigens MUC1(Tn) and MUC1(STn) and the lectin MGL by use of OT. Additionally, we apply the OT based experimental strategy to explore the interaction between a short synthetic polymer carrying GalNAc and MGL. These additional experiments provide information relevant for identifying the chemical groups essential for the observed MUC1—MGL interactions.

## Materials and methods

### Samples

MUC1-IgG Tn, STn and ST samples were produced using wt and mutant CHO cell expression systems as previously described [35, 40]. The molecules contained the extracellular part of human MUC1, including 16 MUC1 tandem repeats. The molar mass of the core polypeptide chain of the MUC1 molecules was 46 kDa. They also carried an IgG domain with MW of about 50 kDa. Each tandem repeat had 5 glycosylation sites, and their average glycosylations, as determined by mass spectroscopy, were: MUC1(Tn) = 3.4, MUC1(STn) = 3.8, and MUC1(ST) = 4.6. The total molecular weights of the glycoprotein constructs were found to be MUC1-IgG-(Tn) = 107 kDa, MUC1-IgG-(STn) = 127 kDa and MUC1-IgG-(ST) = 147 kDa [41]. The glycan decorations on these mucins are summarized in Table 1. α-GalNAc-PEG_3_-NH_2_, referred to as GalNAc-PEG in the following, was obtained from Sussex Research Laboratories Inc. Macrophage galactose/N-acetylgalactosamine (GalNAc) specific lectin (MGL), also known as CLEC10A, was obtained from R&D Systems R&D Systems Inc.Minneapolis, USA.

**Table 1:** Glycan composition.

Sample	Glycan structure
MUC1(Tn)	α-GalNAc-Ser/Thr
MUC1(STn)	α-NeuNAc(2–6)α-GalNAc-Ser/Thr
MUC1(ST)	α-NeuNAc(2–3)β-Gal(1–3)α-GalNAc-Ser/Thr^¤^

NeuAc may be attached to the C6 position of the GalNAc residue [12].

### Covalent attachment of molecules to polystyrene beads

MUC1 molecules, MGL and GalNAc-PEG were immobilized to colloidal polystyrene beads (Spherotech, Lake Forest, Illinois). The immobilization procedure was based on the introduction of a covalent bond between amino groups on the polystyrene beads and carboxyl groups on the molecule to be immobilized, or vice versa, using the water soluble carbodiimide EDC (1-(3-dimethylaminopropyl) -3-ethylcarbodiimide hydrochloride) as a catalyst of the bond formation between the carboxylic acid and amine groups. The immobilization protocol was previously used for immobilization of proteins including mucins onto amine functionalized glass surfaces [42, 43]. When investigating the interaction between MGL and MUC1(Tn), MUC1(STn) or MUC1(ST), the MGL lectins were dissolved in 100 μl aqueous boric acid (50 mM, pH 5.8, referred to as conjugation buffer) at a concentration of 0.1 mg/ml. Amine-terminated polystyrene (nominal diameter 3.07 μm) and EDC were added to this solution to final concentrations equal to 0.03% w/v and 2.5 mg/ml, respectively. The MUC1 molecules were dissolved in 100 μl of the conjugation buffer to a concentration equal to 0.2 mg/ml, and amine-terminated polystyrene beads (nominal diameter 2.01 μm) and EDC were added to final concentrations equal to 0.03% w/v and 2.5 mg/ml, respectively. When investigating the interaction between MGL and GalNAc-PEG_3_-NH_2_, the MGL was immobilized via their carboxylic acid groups onto 2.01 μm amine functionalised polystyrene beads. The concentrations used were equal to 0.1 mg/ml, 0.03% w/v and 2.5 mg/ml for the MGL, polystyrene beads and EDC, respectively. GalNAc-PEG_3_-NH_2_ were immobilized onto carboxylic acid functionalized polystyrene beads (nominal diameter 3.07 μm), using a concentration of GalNAc-PEG_3_-NH_2_, EDC and polystyrene beads equal to 0.5 mg/ml, 2.5 mg/ml and 0.03% w/v, and they were dissolved in 100 μl of conjugation buffer. Unreacted reagents were removed from the functionalized beads by centrifugation (10000 rpm, 4 min), and the beads were re-suspended in aqueous 100 mM Hepes buffer pH 7.2 containing 1 mM MnCl_2_ and 1 mM CaCl_2_. The bead functionalization procedures were carried out at room temperature (20°C). Prior to OT experiments, 3 μl of each of the two functionalized bead solutions that was intended studied was diluted in 50 μl of the Hepes buffer and transferred to the sample chamber of the OT. The calcium dependence of the interactions were investigated by diluting the functionalized bead solutions in either 100 mM Hepes buffer pH 7.2 or in 100 mM Hepes buffer pH 7.2 containing 1 mM MnCl_2_, 1 mM CaCl_2_ and 5 mM EDTA.

### Optical tweezers

Optical tweezer measurements were carried out using the dual beam instrument JPK Nanotracker (JPK Instruments, Berlin, Germany). The sample chambers were made from a circular glass slide, two pieces of double-sided tape and a quadratic coverslip. The circular glass slides used as floors in the sample chambers were pre-coated with bovine serum albumin (BSA, Sigma) (1 mg/ml, 20 min incubation) to reduce adhesion of the functionalized polystyrene beads. The solution containing the functionalized beads was introduced in the sample chamber by capillary forces, prior to sealing the sample chamber and mounting it on the sample stage of the OT. Prior to all measurements, one bead of 2.01 μm in diameter and one bead of 3.07 μm in diameter were identified based on their size using the microscope and captured in separate optical traps. The trap stiffness was determined for each trap prior to each experiment from the power spectra obtained by tracking the 3D Brownian motion of the bead [44]. During the experiments the beads are moved in the x-y plane. Based on the detected displacement of the bead relative to the laser focus as well as the trap stiffness and trap sensitivity, the force withheld by the molecular bond prior to rupture is determined. Performing the calibration procedure 13 subsequent times on the same bead revealed a relative uncertainty in the determination of the trap stiffness of 5.8%. The detection of the bead position relative to the laser beam was based on back focal plane interferometry. The OT instrument used has a force resolution of less than 0.1 pN.

### Observation of forced unbinding of MUC1—MGL interactions using optical tweezers

**Fig 1:**
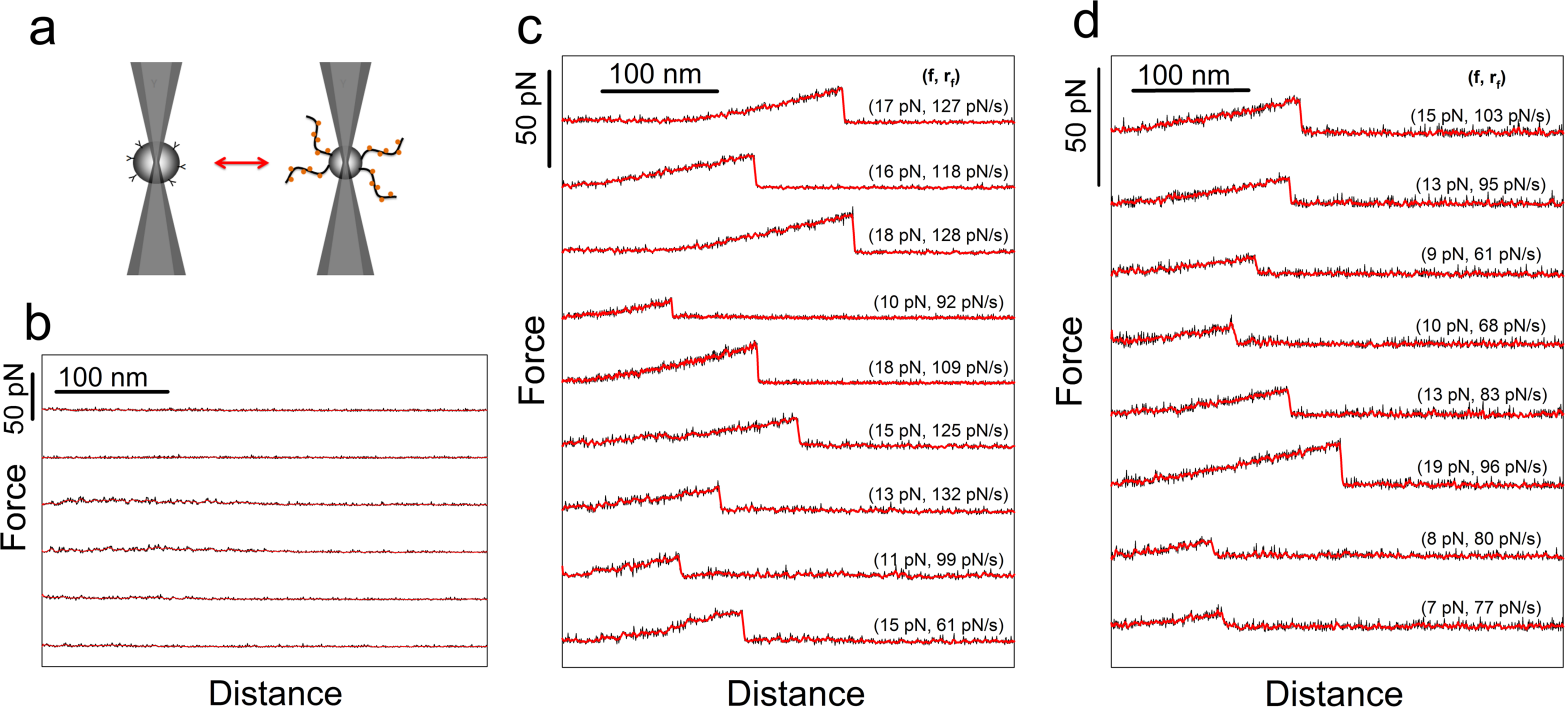
(a) Schematic illustration of two optically trapped beads functionalized with MUC1 and MGL respectively. During one approach–retract cycle, the beads are brought in contact and then apart. If an intermolecular bond is formed between a MUC1 molecule and an MGL, a force will be applied to this bond during bead retraction, and ultimately lead to its rupture. b-d: Examples of typical curves obtained when repeating the approach–retract cycle explained in (a) for experimental series of (b) MUC1(ST)–MGL, (c) MUC1(Tn)–MGL and (d) MUC1(STn)–MGL, respectively. Raw (black) and smoothed (red) data are shown. The smoothed curves were used in the further data analysis. For each observed rupture event, the loading rate *r*_*f*_ was determined based on the slope of the force curve prior to bond rupture, while the height of the jump reflects the magnitude of the unbinding force *f*.

The experiments were carried out as outlined in Fig 1A. A MGL-functionalized polystyrene bead was trapped in one of the two optical traps of the dual trap system, and a MUC1 or GalNAc-PEG functionalized bead was trapped in the other. The distance separating the two traps was then reduced until the two polystyrene beads were in contact and pushed each other slightly out of the laser focus, observed as an increase in the force acting on the beads. The beads were left in contact for 0.8 s before increasing the bead separation distance. When increasing the inter-bead distance, intermolecular interactions between the mucins or mucin analogues and MGL, if formed, were broken due to the applied force. Prior to bond rupture, the beads are displaced relative to the center of the optical trap in proportion to the force acting on the beads. Repeated approach—retract cycles were carried out with bead separations in the range 1.5–3 μm.

### Analysis of intermolecular bond rupture events

The bond strength and the corresponding force loading rate applied to the bond just prior to rupture were determined for each observed rupture event based on the magnitude and slope of the force jump, respectively. The raw data sampled at 2.1 kHz at the retraction speed of 1 μm/s, were smoothed using a 6 datapoint moving average and analyzed with respect to unbinding events. The occurrence of the force unbinding events were determined based on differentiating the smoothed raw data to make the identification of force jumps more easy to distinguish from signals from the noise. The procedure employed to determine the force loading rate uses a linear approximation of the increase in force just prior to the unbinding event, and is previously reported [45]. The data segments of the force-traces just prior to the unbinding events are selected based on balancing the suppression of effect of noise in the data while conforming to the linear approximation. The experimentally determined energy landscapes of the macromolecular interactions were interpreted based on the theoretical framework [46–54] outlined in the following.

According to the model first proposed by Bell and later elaborated by Evans and coworkers the dissociation rate related to the transition from a bound to an unbound free state is for a molecular pair dependent on the applied force [49, 50, 54]. Key parameters appearing in this model include x_β_ which is defined as the thermally averaged distance from the bound complex to the transition state projected along the direction of the applied force and k_B_T, the thermal energy. Consequently, the rate of dissociation under a constant loading force *f*, *k*_*off*_*(f)*, exponentially increasing with the force:

$$k_{off}(f)=k_{off,0}\exp\left(\frac{x_{\beta }f}{k_{B}T}\right)$$ (1)

the probability density *P(f)* for observing a bond rupture between a molecular pair at the force *f* subjected to constant force loading rate *r*_*f*_ predicted by the Bell-Evans assumption is:

$$P(f)=k_{off,0}\exp\left(\frac{x_{\beta }f}{k_{B}T}\right)\exp\left[\frac{k_{off,0}k_{B}T}{x_{\beta }r_{f}}\left(1-\exp\left(\frac{x_{\beta }f}{k_{B}T}\right)\right)\right]$$ (2)

when the applied force along the unbinding pathway exceeds the force *f*_*β*_ governed by the distance *x*_*β*_, i.e., *f*_*β*_ *= k*_*B*_*T/x*_*β*_ an exponential increase in the most likely unbinding force, f^*^, is predicted [49, 50, 54].

$$f^{*}=f_{\beta }\ln\left(r_{f}/r_{f}^{0}\right)$$ (3)

parameter *r*_*f*_ is the actual force loading rate, and *r*_*f*_^*0*^ a thermal scale for loading rate, *r*_*f*_^*0*^ = *f*_*β*_*/t*_*0*_ where *t*_*0*_ is the inverse of the transition rate. Parameters characterizing the interactions between the mucins and lectin were extracted from the data generated by the OT as follows. The set of data of f versus *r_f_* for each type of macromolecular pairs were divided into intervals with equal range of Ɗln(*r_f_*) for the intervals. The mean value of rf and spread represented by the standard deviation of the set of the data within each interval were estimated and a histogram was estimated. The most probable unbinding force f* within each interval was estimated using a non-linear fit of P(*f*) (Eq 2) to histograms centered around a mean force loading rate. Parameter *x_β_* was estimated by fitting the linearized version of Eq 3 to the estimated mean *r_f_* and f* as outlined above. The uncertainties of *x_β_* were estimated based on the uncertainty of the slopes determined in the fitting to the linear version of Eq 3. Estimates of k_*off, 0*_ were determined from the estimated intercept in the fit of the linear version of Eq 3 from the procedure used to estimate *x_β_*. A constrained fit of P(f) keeping the *x_β_* parameter constant was used to guide an eventual splitting of the f** vs *r_f_* data into regions, each conforming more closely to the behavior predicted by Eq 3, e.g., representing barriers with their particular parameters, than when assuming one barrier.

## Results

### Observations of unbinding events for single molecular pairs of MUC1 and MGL

The experiments were carried out by trapping a MGL-functionalized polystyrene bead in one of the two optical traps of the OT setup and a MUC1 or GalNAc-PEG functionalized bead in the other (Fig 1A). When bringing two polystyrene beads in contact and then increasing the inter bead distance, any intermolecular interactions between the mucins or mucin analogues and MGL, were broken due to the applied force. Frequent force jumps, reflecting the rupture of intermolecular interactions, were observed in the force curves when MGL interacted with either MUC1(Tn) or MUC1(STn), but not when allowing MGL to interact with MUC1(ST) (Fig 1).

**Fig 2:**
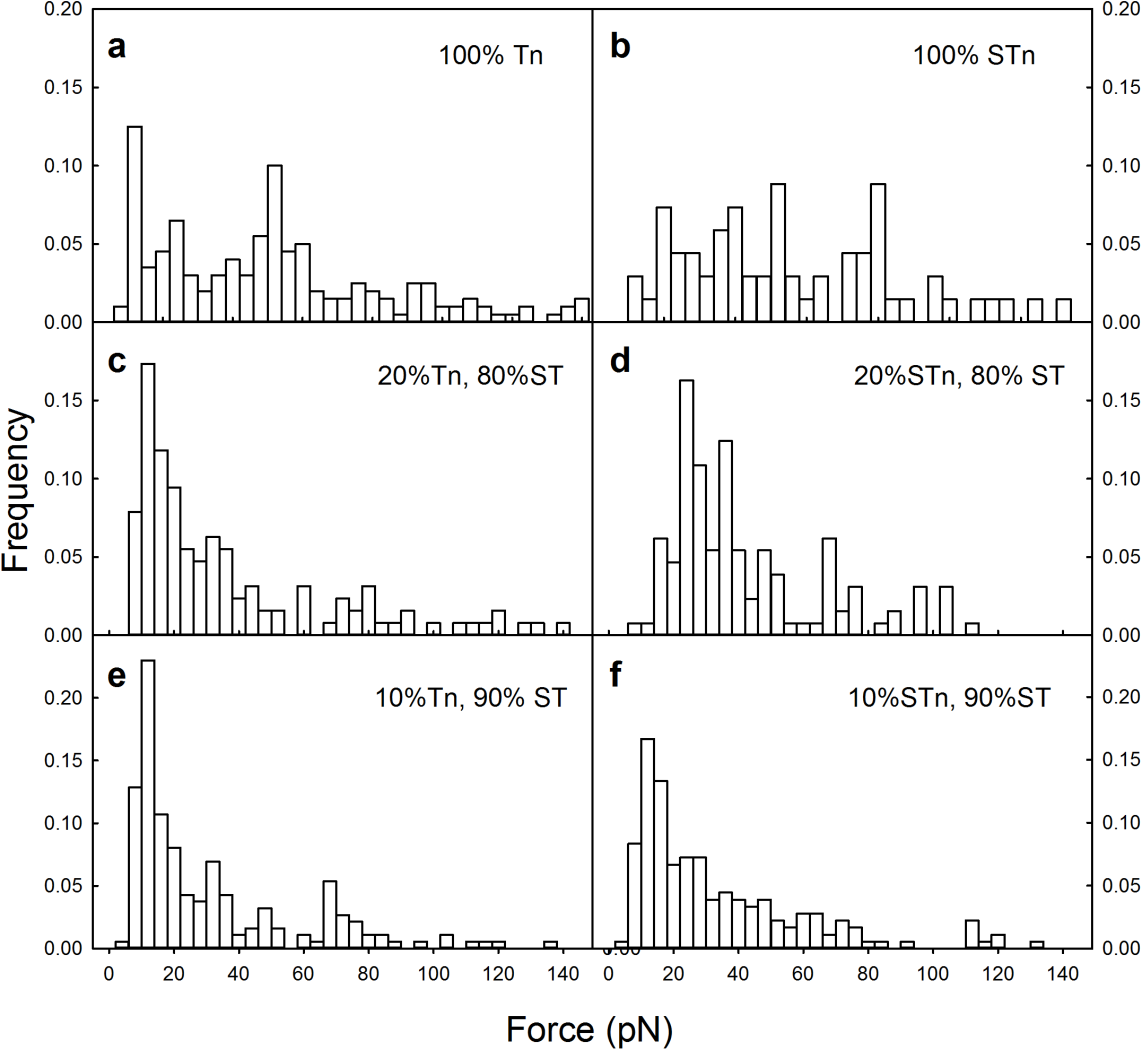
Distributions of intermolecular rupture events between MUC1 mucins and MGL obtained in optical tweezer experiments using polystyrene beads functionalized with MUC1(Tn) (a) and MUC1(STn) (b), mixture of 80% MUC1(ST) and 20% MUC1(Tn) (c) or MUC1(STn) (d) or a mixture of 90% MUC1(ST) and 10% MUC1(Tn) (e) or MUC1(STn) (f). For each of the six experimental series around 150 forced rupture events were collected and included in the analysis.

**Fig 3:**
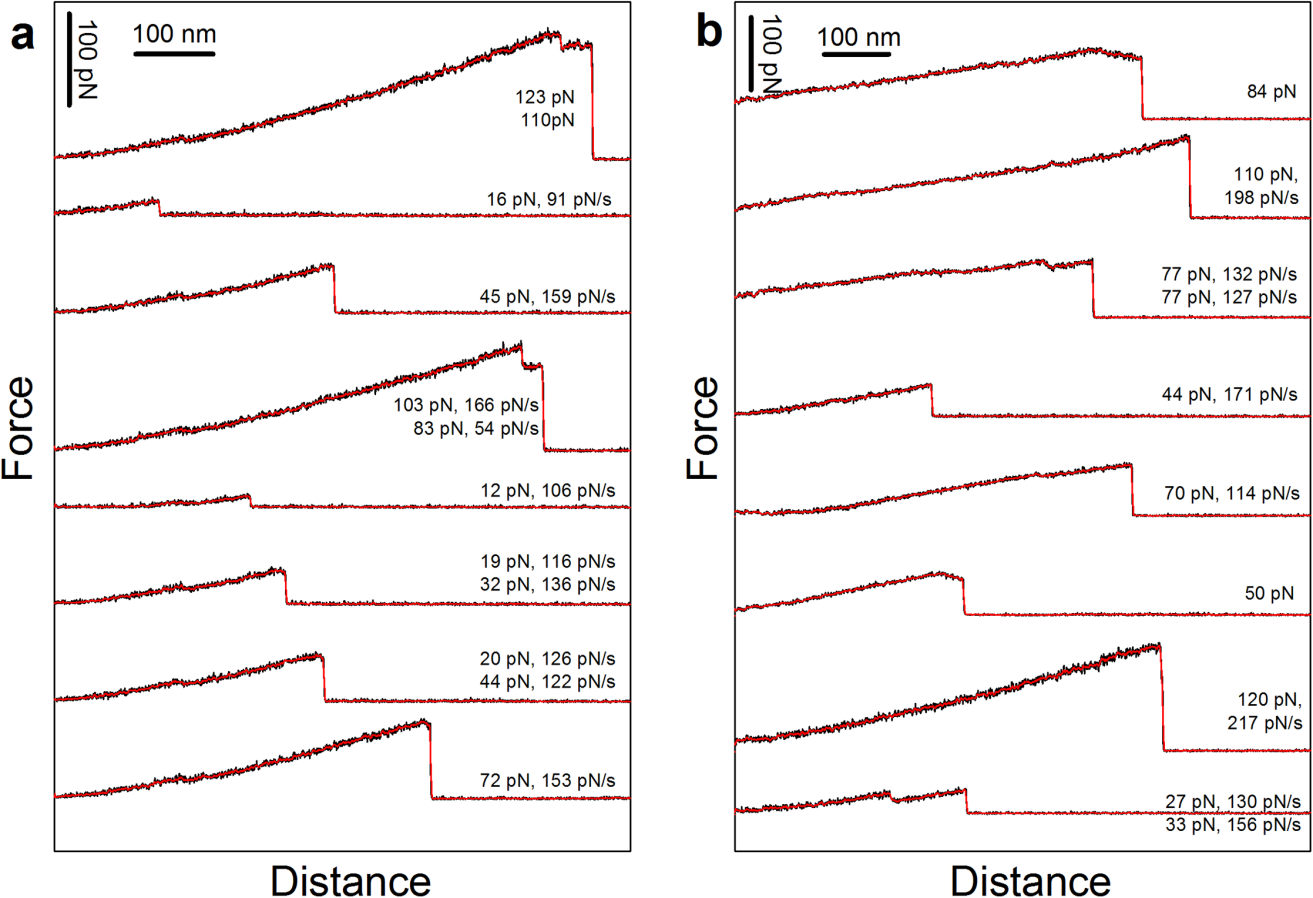
Examples of typical force versus inter-bead distance curves obtained for (a) MUC1(Tn)–MGL interactions and (b) MUC1(STn)–MGL interactions, respectively. Raw (black) and smoothed (red) data are shown. The curves were obtained when using the experimental conditions explained in Fig 2A and 2B, respectively. For some of the curves successive rupture events can be identified (panel a curve 1, 4, 6 and 7 from top, panel b curve 3 and 8 from top) whereas in others the high rupture forces and/or abnormal shape of the force versus distance trace prior to rupture are indications of the multiplicity of the rupture events. For some of the rupture events presented a negative increase in the force prior to rupture, i.e. negative force loading rates, are observed (panel a curve 1 from top, panel b curve 1 and 6 from top). This is an abnormal behavior that can be explained by a multiplicity of bonds being successively broken. For these observations the force loading rate is therefore not indicated in the figure.

The histogram of the rupture forces for the MUC1(Tn)–MGL or MUC1(STn)–MGL pairs revealed a large spread (Fig 2A and 2B). Such broad distributions are observed for macromolecular pairs in direct force unbinding assays having a high probability for multiple interactions. The existence of multiple interactions in these experimental series is confirmed by the appearance of the force curves (Fig 3A and 3B). Typical force curves obtained for MUC1(Tn)–MGL ((Fig 3A) and MUC1(STn)–MGL (Fig 3B) obtained when using the experimental conditions explained in Fig 2A and 2B display successive rupture events and/or high rupture forces. Due to the observed inability of MUC1(ST) to interact with MGL, these molecules were in later experimental series used as non-interacting spacer molecules between the interacting MUC1(Tn) and MUC1(STn) molecules on the beads studied using OT. Experimentally, surfaces displaying MUC1(ST) in addition to the MUC1(Tn) or MUC1(STn) were obtained by mixing MUC1(ST) and MUC1(Tn) or MUC(STn) in the conjugation buffer. A mixing ratio of 80% (0.08 mg/ml) MUC1(ST) and 20% (0.02 mg/ml) MUC1(Tn) or MUC1(STn) in the conjugation buffer yielded a significant reduction in the high force tail of the force distribution (Fig 2C and 2D). For MUC1(Tn) a narrow distribution of rupture forces was observed, with a most probable rupture force located at 12 pN (Fig 2C). For MUC1(STn) a broader distribution was observed (Fig 2D). Further increasing the fraction of the MUC1(ST) to 90% of the total content of glycoprotein in the solution during the conjugation step did not significantly influence the distribution of interaction forces observed for MUC1(Tn) (Fig 2E). However, in the case of STn, this reduction resulted in a more narrow distribution of rupture forces and a reduction in the most probable rupture force from 24 to 12 pN (Fig 2F). Based on these observations, a concentration equal to 0.01 mg/ml MUC1(Tn or STn) and 0.09 mg/ml MUC1(ST) were identified as the optimal concentrations and were in this study used as basis for assessment of single-molecular pair interactions. The hypothesis that the high rupture forces observed when using the experimental conditions underlying the histogram distributions presented in Fig 2A and 2B are due to multiple MUC1-MGL interactions are supported the signatures observed in the force curves obtained when using these experimental conditions (Fig 3A and 3B). These force curves contain increased probability for successive rupture events compared to what is obtained when decreasing the density of the interacting molecules (Fig 1C and 1D, obtained at the experimental conditions explained in Fig 2E and 2F, respectively).

### Dynamic force spectroscopy of MUC1–MGL interactions

The force–distance curves obtained for the MUC1(Tn)—MGL interaction (Fig 1C) allowed identification of 1268 force jumps. For the MUC1(STn)–MGL interaction (Fig 1D) 1648 force jumps were collected. For each of these force jumps, the rupture force and the loading rate were determined.

**Fig 4:**
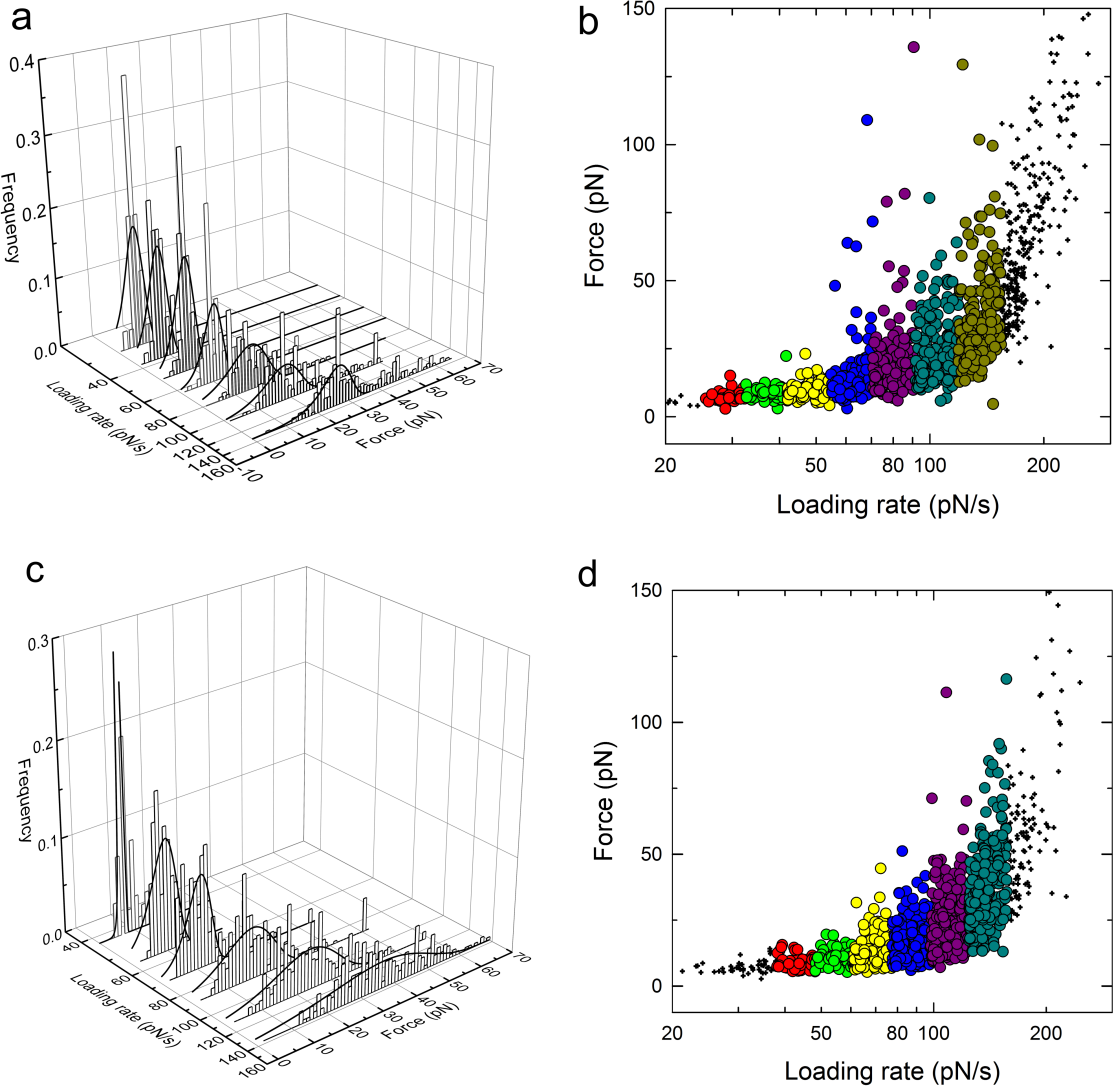
Distribution of experimentally determined MUC1(Tn)—MGL (a and b) and MUC1(STn)—MGL (c and d) rupture forces at increasing force loading rates. The data are collected from force curves obtained using a retraction speeds equal to 1 μm/s. The loading rate *r*_*f*_ acting at a molecular bond was determined for each force jump from the slope *Δf/Δt* before each observed bond dissociation event. Based on the determined loading rate, subgroups were defined within the continuous distribution of observations. (a and c): 3-dimensional plot revealing the histogram distributions of experimentally determined rupture forces contained within each of the predefined intervals of loading rates as a function of increasing force-loading rate. The continuous line depicts the fit of P(f) (Eq 2) to the histograms. (b and d): 2-dimensional plot revealing the distribution of rupture events as a function of increasing force-loading rate. The color-coding (large symbols) depicts the groups of data for the histogram analysis, whereas the smaller symbols depict observations where subgroups where not sufficiently large to apply the histogram analysis.

**Fig 5:**
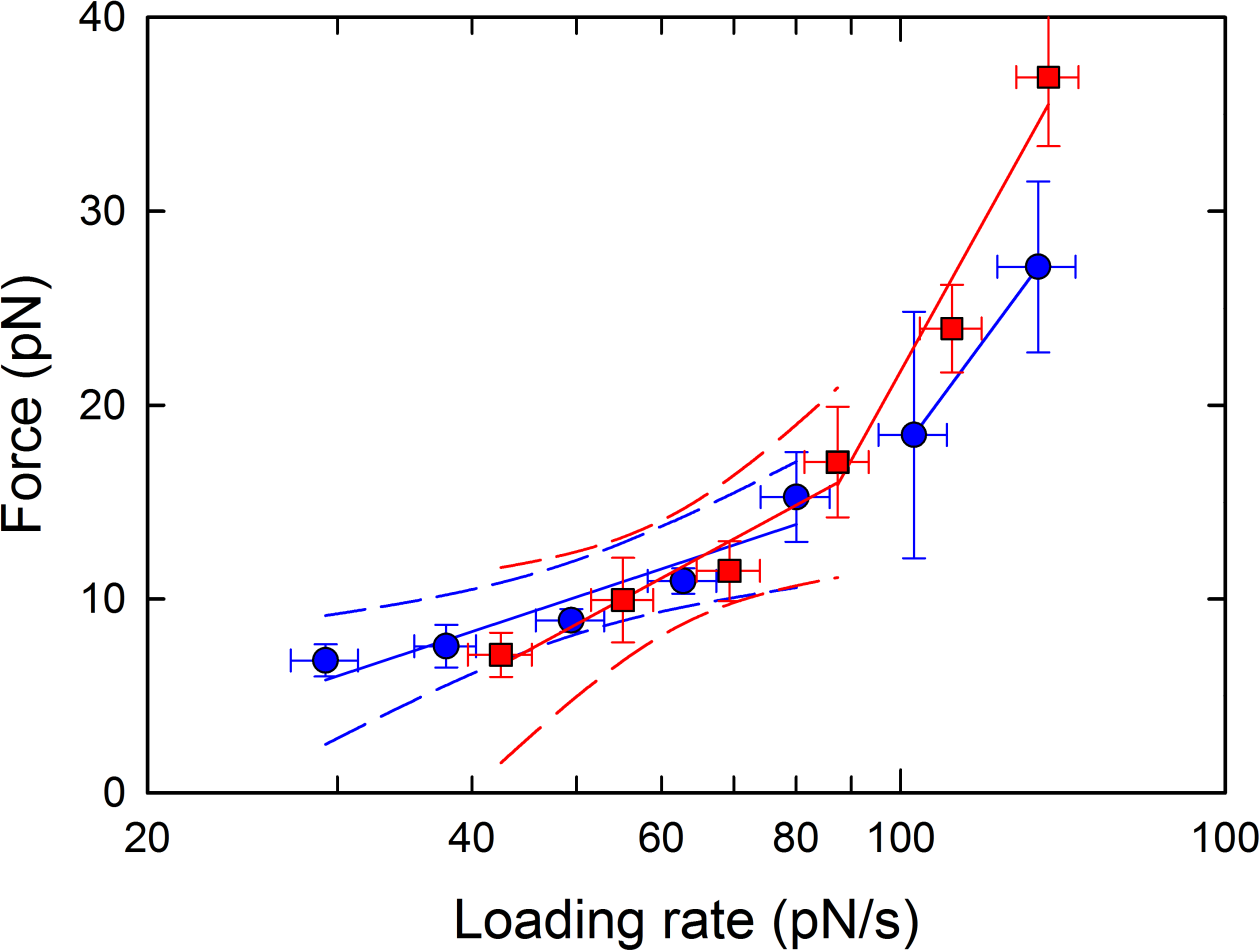
Dynamic force spectra for the MUC1(Tn)–MGL (blue points) and for MUC1(STn)–MGL (red points). The most probable unbinding forces f* (large points) were determined from the fit of Eq 2 to the histograms depicted in Fig 4A and 4C, and were plotted versus increasing force loading rate.The data points are depicted with symbols for the mean values and error bars representing the spread of *r*_f_ in the intervals (standard deviation) and standard deviation of f* being the uncertainty of the estimates. The continuous lines depict the result of a linear regression performed for the data contained within each linear regime of the dynamic force spectrum. The discontinuous lines depict the 95% confidence intervals of the linear regressions.

The data contained in the dynamic force spectrum obtained for the MUC1(Tn)–MGL interaction were grouped into 7 subgroups along the axis of increasing mean loading rates, from an average loading rate equal to 29 pN/s for the first interval to 134 pN/s for the last interval (Fig 4A and 4B). The probability density function of unbinding under external force, P(f) (Eq 2) was fitted to the distribution of unbinding forces contained in subgroup, using the parameters k_*off, 0*_, reflecting the lifetime of the interaction, and x_*β*_ as fitting parameters. The most probable rupture force f* was for each subgroup determined based on the peak in the probability function. Table 2 summarizes the number of observations contained in each subgroup as well as the estimates obtained for k_*off, 0*_, x_*β*_, the average loading rate *r*_*f*_ and the most probable rupture force f*. For the interval characterized by an average loading rate *r*_*f*_ equal to 29 pN/s, unbinding forces ranging from 5 to 14 pN were observed, with a most probable value equal to 6.8 pN. For this interval, x_β_ was determined to 0.51±1.1 nm, and k_*off, 0*_ to 2.0 s^-1^. The most probable unbinding forces increased with increasing force loading rate and f^*^ = 27 pN was determined for the subgroup with average loading rate *r*_*f*_ = 134 pN/s. For this interval, x_β_ was estimated to 0.12 nm, and k_*off, 0*_ to 1.9 s^-1^ (Table 3). The force jumps observed for the MUC1(STn)—MGL interaction were divided into 6 subgroups (Fig 4C and 4D). The estimates obtained for the key parameters describing this interaction are presented in Table 3. For the lowest loading rate range (mean loading rate 43 pN/s), the most probable rupture force f^*^ was equal to 7.1 pN, x_β_ was determined to 0.31±0.1 nm, and k_*off, 0*_ was determined to be 3.3 s^-1^. For the subgroup with the highest mean loading rate, 137 pN/s, the most probable unbinding force was determined to be f^*^ = 37 pN, x_β_ was determined to be 0.09 nm, and k_*off, 0*_ was determined to be 1.8 s^-1^ (Table 3). The dynamic force spectra obtained for these two interactions (Fig 5) are also similar. The 95% confidence intervals of the fit of f* vs ln (*r*_f_) to the linear version of Eq 3, indicating large overlap of the domains, suggest that parameters of the unbinding barriers at lowest range of *r*_f_ are similar for the MUC1(Tn)-MGL and MUC1(STn) interactions.

**Table 2:** Estimated parameters characterizing the energy landscape of the MUC1(Tn)—MGL interaction.

Interval	Number of observations	r_f_ (pN/s)	f* (pN)^a^	x_β_ (nm)	k_*off, 0*_ (1/s)
1	37	29±2	6.8±0.8	0.51±0.11	2.0^c^
2	71	38±2	7.6±1.1	0.51±0.11	2.3^c^
3	150	49±3	8.9±0.6	0.51±0.11	2.3^c^
4	184	63±4	11.0±0.6	0.51±0.11	1.9^c^
5	218	80±6	15.3±2.3	0.51±0.11	1.5^c^
6	291	103±7	18.5±6.4	0.12^b^	^d^
7	317	134±11	27.0±4.4	0.12^b^	^d^

a) f* designates the most probable unbinding force of the intermolecular interaction.

b) The relative uncertainty in the estimate of *x*_β_ in this upper range of *r*_f_ cannot be estimated due to the number of datapoints.

c) The uncertainty in *k*_off,0_ is in the order of half an order of magnitude (on the logarithmic) scale when estimated based on fit to the linearized version of Eq 3.

d) Not determined due to limited data.

**Table 3:** Estimated parameters characterizing the energy landscape of the MUC1(STn)—MGL interaction.

Interval	Number of observations	r_f_ (pN/s)	f* (pN)^a^	x_β_ (nm)	k_*off, 0*_ (1/s)
1	83	43±3	7.1±1.1	0.31±0.1	3.3^c^
2	130	55±4	9.9±2.2	0.31±0.1	3.0^c^
3	207	69±5	11.4±1.5	0.31±0.1	2.9^c^
4	422	87±6	17.0±2.9	0.09^b^	^d^
5	503	112±7	24.0±2.3	0.09^b^	^d^
6	303	137±9	36.9±3.6	0.09^b^	^d^

a) f* designates the most probable unbinding force of the intermolecular interaction.

b) The relative uncertainty in the estimate of *x*_β_ in this range of *r*_f_ is larger than for the lower range of *r*_f_.

c) The uncertainty in *k*_off,0_ is in the order of half an order of magnitude (on the logarithmic) scale when estimated based on fit to the linearized version of Eq 3.

d) Not determined due to large uncertainties

**Fig 6:**
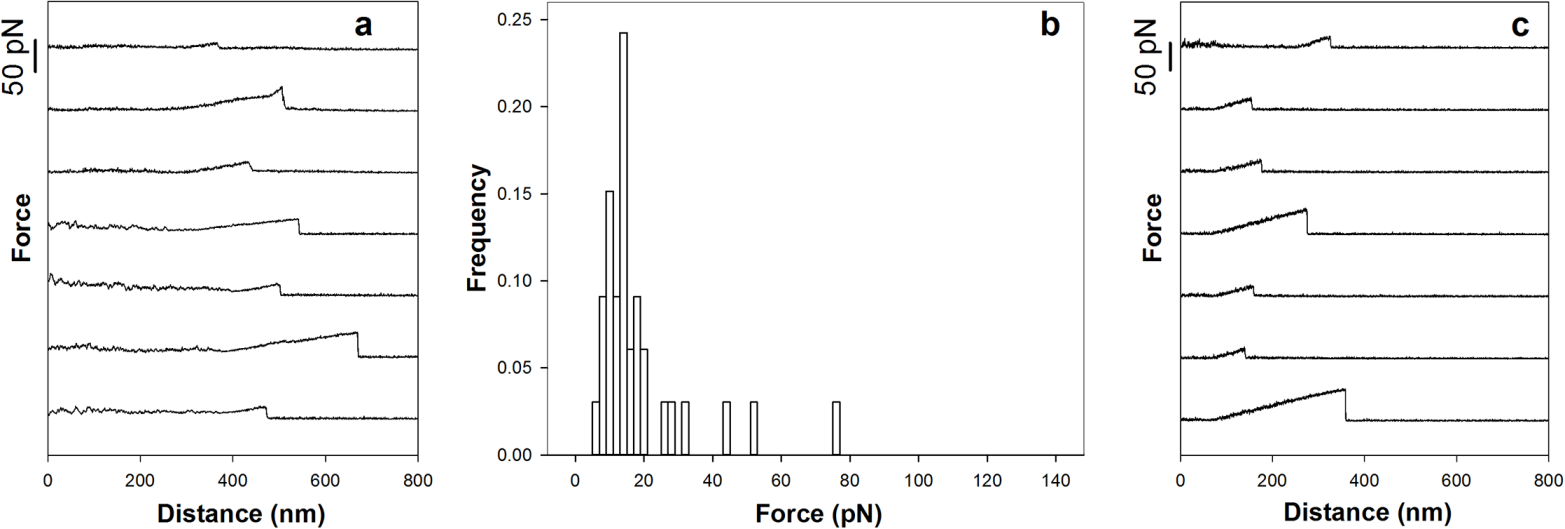
Optical tweezer characterization of the MUC1(Tn)–MGL interaction in buffer containing Ca^2+^ chelator. (a) Force versus distance curves obtained after adding EDTA to the Ca^2+^ containing Hepes buffer that filled up the sample chamber of the OT instrument. (b) Distributions of intermolecular rupture events between MUC1 mucins and MGL determined under the experimental conditions described in (a). The histogram distribution is based on 33 observed rupture events. (c) Force versus distance curves obtained when investigating the interaction between MUC1(Tn) and MGL in a sample chamber filled with a Hepes buffer not containing Ca^2+^ ions.

In order to investigate the potential Ca^2+^ dependence of the interactions, OT experiments were performed prior to and after adding EDTA to the 100 mM Hepes buffer pH 7.2 containing 1 mM MnCl_2_ and 1 mM CaCl_2_. Frequent force jumps were observed both prior to and after adding EDTA (Fig 6A). The distribution of the rupture forces (Fig 6B) revealed a similar interaction strength as observed for the MUC1(Tn)–MGL interaction when investigated in the Hepes buffer prios to addition of EDTA (Fig 2E). Also in experimental series performed using 100 mM Hepes not containing Ca^2+^, force jumps were observed (Fig 6C).

### Observation of interaction between MGL and GalNAc-PEG

**Fig 7:**
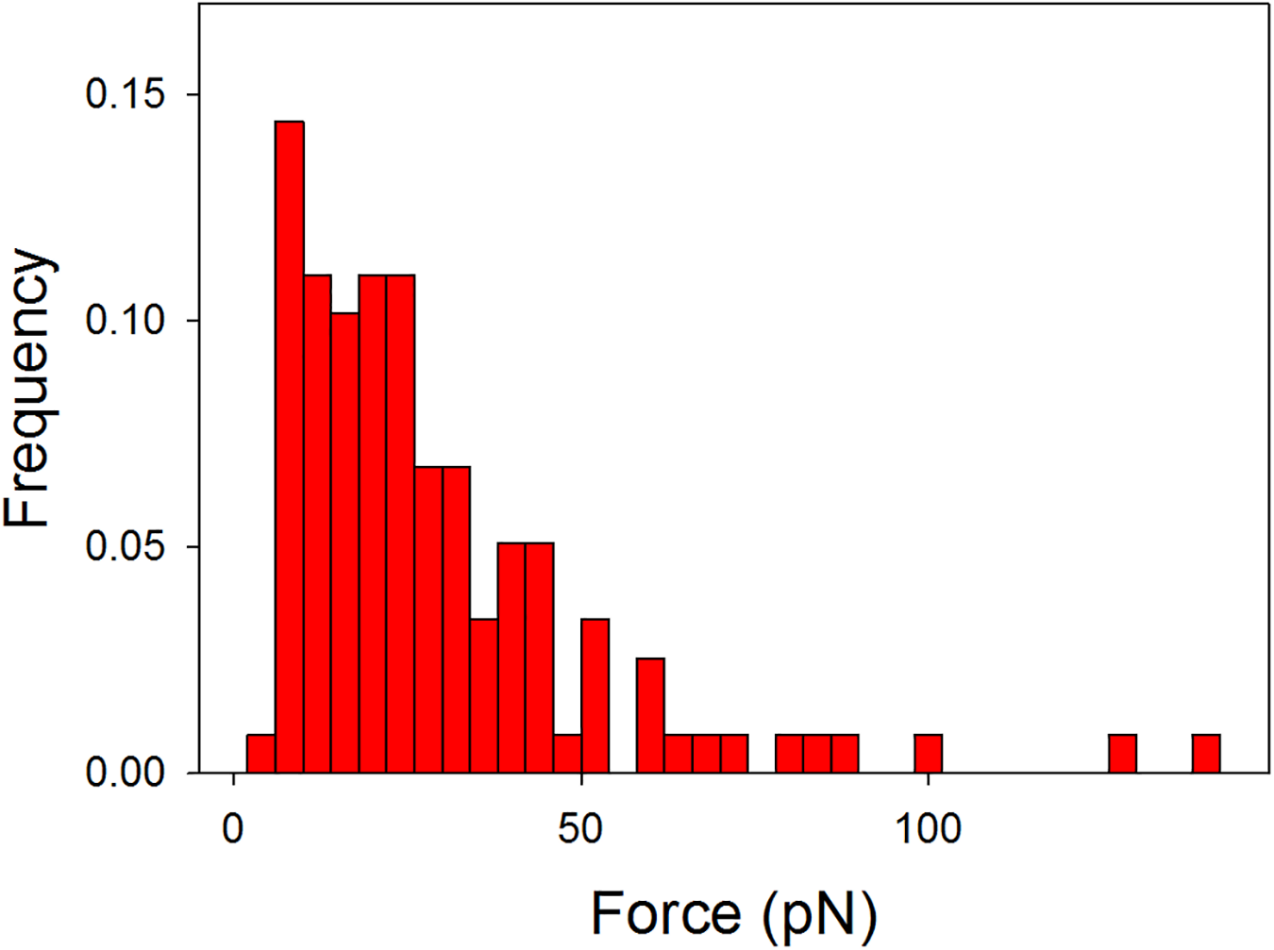
Histogram presenting the strength of the unbinding forces observed for the GalNAc–MGL interaction.

To further study the role of the sugar residue of MUC1(Tn) involved in the MGL binding, polystyrene beads functionalized with the α-GalNAc-PEG_3_-NH_2_ were prepared. When bringing these beads in contact with polystyrene beads functionalized with MGL, signatures of rupture of intermolecular bonds were observed upon bead separation. Based on the obtained force–distance curves 35 rupture events were identified. The distribution of rupture forces observed for these interactions are presented in Fig 7.

## Discussion

Some of the challenges faced when studying carbohydrate—protein interactions are related to their inherent low strength and multivalency. These challenges have hampered the progress in the emerging field of glycomics, and this field is therefore expected to benefit from the application of new methodologies. Single-molecule manipulation techniques have evolved rapidly over the past decades and are finding an increasing number of applications. However, whereas the number of studies in which AFM is used to quantify intermolecular interactions increased rapidly, OT is still rarely used in such studies. Despite this, the low force range attainable with OT makes this probe a powerful tool for quantifying weak intermolecular interactions. In the current study, OT is used to investigate the interaction between the mucin MUC1 and the lectin MGL. The results obtained reveal that intermolecular bonds form between MGL and MUC1(Tn) or MUC1(STn), but not with MUC1(ST) (Fig 1). The observations presented in this paper are thus compatible with a hypothesis where MGL specifically binds to cancer-associated mucins, as previously proposed [24–26].

When quantifying the strength of single molecular bonds, a low probability of observing an interaction event is essential since this assures a high probability that the rupture events observed reflect the rupture of single molecular bonds. In the current study, good control of the surface density of MUC1(Tn) and MUC1(STn) was obtained by adding the non-interacting MUC1(ST) molecules to the solution used for surface functionalization. When applying this strategy the density of the interacting molecules, and thus the tendency for multiple interactions, was efficiently controlled. An additional benefit of the approach used is the reduction in the possibility for non-specific interactions between areas of the polystyrene beads not displaying MUC1 molecules. The contribution of such non-specific interactions in the data set would otherwise contribute with noise and hamper a correct determination of the interaction force between single molecular pairs of MUC1 and MGL. Following optimization of the immobilization procedure to yield mainly single-molecular pair unbinding events, the most probable rupture force was determined to 12 pN (Fig 2) for both MUC1(Tn)—MGL and MUC1(STn)—MGL interactions. The number of reported OT based quantitative studies of carbohydrate-protein interactions is limited. However, the interaction of the *H*. *pylori* adhesin BabA with the antigen Lewis b has been quantified using OT [7], and the rupture events were observed to be centered at multiples of 12.5 pN. Due to an observed high probability for 25, 50 or 75 pN, with lower probability for the intermediary strengths, the authors concluded that the strength of the BabA-Leb interaction was equal to 25 pN [7].

The parameters x_β_ and k_*off, 0*_ were determined for both the MUC1(Tn) and the MUC1(STn) interactions with MGL (Tables 2 and 3). These parameters provide information related to the shape of the energy landscape of the intermolecular interactions as well as the lifetime of the interaction, respectively. Unfortunately, these parameters were not determined in the previous study of BabA–Lewis b interactions. In the current study, very similar lifetimes were determined for the two interactions (Tables 2 and 3). The average lifetimes of the interaction, reflected by the parameters k_*off, 0*_, were for the inner barriers determined to 0.5 s and 0.3 s for the MUC1(Tn)—MGL and MUC1(STn) systems, respectively. These lifetimes are in the same range as previously determined for other carbohydrate–protein interactions using AFM [42, 43] and OT [55]. As expected it is shorter than the lifetime of antibody-peptide interactions [56] as well as the interaction between cell-surface sulfatase Sulf1 and glycosaminoglycans [57]. Despite the similarity of both the binding strengths and the lifetimes of the MUC1(Tn) and MUC1(STn) interactions with MGL, a slightly lower x_β_ value was determined for the MUC1(STn)–MGL system (Tables 2 and 3). The values obtained in the current study, being in the range 0.09–0.51 nm, are comparable in size to the values determined in previous single molecule studies on related systems. Previous AFM based quantitative studies of the interaction between porcine submaxillary mucine (PSM) interacting with the lectin SBA, gave x_β_ values in the range of 0.05–0.12 nm, decreasing with increasing force loading rate [43]. The direct comparison of data obtained by OT and AFM need to take into account the differences in loading rates realized using these techniques. AFM provides data in a higher loading rate range compared to OT. Provided the molecular interaction possesses an inner energy barrier in the energy landscape, the characterization by AFM may yield lower x_β_ values [49]. The similarity of the rupture force and lifetimes of the MUC1(Tn) and MUC1(STn) interaction with MGL (Tables 2 and 3) is consistent with the main conclusions drawn based on previous NMR data, which indicated a similar binding mode for the Tn and the sialylated Tn antigen when interacting with MGL [30]. The NMR data indicated that the N-acetyl group and the H-2, H-3 and H-4 protons of the GalNAc residue made the major contribution to the interaction [30]. However, the NMR data also indicated a slightly lower affinity of the STn antigen for MGL compared to the Tn antigen [30]. In the current study small variations between the two interactions were also observed: the parameter x_β_ was slightly lower for the MUC1(STn)–MGL relative to the MUC1(Tn)—MGL interaction. This might indicate a slightly shorter separation distance between the MUC1(STn) and the MGL when bound to each other. However, due to the relatively small variations observed combined with the experimental challenges related to the determination of this parameter clear conclusions should not be drawn based on this difference. The slightly lower affinity of the STn antigen for MGL observed using NMR but not when studying the interaction using OT might be due to the fact that in the NMR study, mucin analogs in which the glycans were linked to a single serine unit were used. It can at this stage not be ruled out that the amino acid portion of the glycoprotein somewhat influences on the properties of the interaction, as recently proposed [58].

The experimental data revealed no Ca^2+^ dependence of the MUC1(Tn)–MGL interaction (Fig 6). Ca^2+^ dependent binding is observed for several other lectin–glycan interactions [59, 60]. The Ca^2+^ dependence is also previously investigated for carbohydrate–carbohydrate interactions, where it is observed for some glycans [61], whereas in other studies no such dependence [62], or only a weak dependence, is reported [63]. In a previous study of Ca^2+^-dependent cell adhesion, relatively strong adhesive bonds formed also in calcium-free artificial seawater [64]. The rupture forces were of the same order of magnitude as those obtained for the same molecule in the presence of 10 mM Ca^2+^. However, more detailed analysis of the unbinding process revealed that the lifetime of the bound complex was longer in the presence of Ca^2+^. Thus, based on the AFM force probe observations, the difference in lifetime, and not the magnitude of the unbinding force, was suggested to explain the Ca^2+^ dependence reported for this system using other experimental techniques. In the present study the investigation of the Ca^2+^ dependence of the interaction was not the main scope, and the determination of the lifetime of the interaction could not be performed in a reliable way based on the limited number of force versus distance curves obtained in the present study for the MUC1(Tn)–MGL interaction. However, similar lifetime parameters were determined for the MUC1(Tn)–MGL and the MUC1(STn)–MGL interaction (Tables 2 and 3). The potential importance of the bond lifetime will thus not influence on the comparison of these two systems. Also other studies point to the importance of the lifetime as well as the on rate for bond formation when studying intermolecular bonds. The values of k_on_ and k_*off, 0*_ as determined using AFM has also previously been reported not to correspond with the determined K_D_ using thermodynamic methods. One example of such discrepancy is the antibiotic vancomycin interacting with its target in *Staphylococcus aureus* that has been characterized by AFM [65], Isothermal calorimetry [66], affinity capillary electrophoresis [67] and competitive titration methods [68]. The value of K_D_ estimated based on the AFM observations was 3–6 orders of magnitude from the range of bulk solution values. Such a discrepancy might be related to the conditions inherent in conventional single molecule force spectroscopy, which include a suboptimal sampling of slowly formed bonds due to the limited time available for bonds to be formed.

Quantification of the intermolecular rupture force between the PEG based mucin analogue carrying GalNAc and MGL revealed that the GalNAc unit binds to the MGL even when not attached to a polypeptide backbone (Fig 7). This is in accordance with previous observations of non-glycosylated MUC1 peptides, which revealed that the sugar residue is essential for MGL binding [69]. GalNAc specific lectins have been shown to bind to mucin mimetic glycopolymers displaying GalNAc attached to synthetic polymer backbones [70]. The results presented in the present paper extend the previous knowledge by affording quantitative information related to the strength of these interactions. The results indicate that the rupture force (Fig 7) is similar to this observed for the MUC1(Tn)–MGL interaction (Fig 2). Quantitative data related to the glycan–lectin interaction, as provided in the present paper, complements the information obtained by other experimental tools and illustrates that the force probe approach is an interesting supplement in studies aiming at revealing the binding capabilities of glycans or other biologically important molecules.

The OT based identification of the molecular groups involved in the MUC1 –MGL interactions, as presented in the current paper, and its consistency with previously published data related to the MUC1 –MGL interactions, demonstrate the strength and reliability of the OT based approach in studies of such weak intermolecular interactions. It is therefore to be expected that future studies using this methodology will contribute with new insight related to a broad range of molecular binding partners, including but not restricted to the weak glycan–lectin interactions, and their functions in cellular systems.

## Conclusions

The low force range attainable with OT makes this force probe well suited for studies of weak intermolecular interactions. We have used OT to provide further evidence of the interactions occurring between MGL lectins and the cancer-associated antigens mucins MUC1(Tn) and MUC1(STn). Since both of these structures are expressed in human tumors, this interaction is a likely mechanism explaining how macrophages or dendritic cells expressing MGL lectins may recognize tumor cells expressing these glycans. The interaction strength increased from 6 to 37 pN over the loading rate interval from 29 to 137 pN/s, and no significant differences in binding strength was observed between the two mucins studied. The experimental data obtained related to the Ca^2+^ dependence are considered consistent with reported Ca^2+^ dependent thermodynamics if taking into consideration the limited access to the kinetics in the OT approach. The observed absence of interactions observed between MGL and MUC1(ST), a structure expressed by breast carcinoma cells as well as by normal cells, points to the specificity of the MUC1(Tn) and MUC1(STn) MGL interaction. The results also demonstrate that MGL is able to bind the monosaccharide GalNAc, the carbohydrate moiety of MUC1(Tn), existing in a sialylated version in MUC1(STn), with comparable binding strength as the MUC1(Tn)—MGL and MUC1(STn)—MGL interactions. These observations are consistent with the interpretation that the GalNAc residue is essential for the MUC1(Tn) and MUC1(STn) interactions with MGL. The consistency between the conclusions obtained here and those previously reported related to the MGL–MUC1 interactions validate the proposed OT based approach for studies of a broad range of molecular interactions, including the weak glycan–protein interactions.
