# Correction: Interactions between the breast cancer-associated MUC1 mucins and C-type lectin characterized by optical tweezers

**DOI:** 10.1371/journal.pone.0190814

**Published:** 2018-01-02

**Authors:** Soosan Hadjialirezaei, Gianfranco Picco, Richard Beatson, Joy Burchell, Bjørn Torger Stokke, Marit Sletmoen

## Notice of Republication

This article was republished on August 8, 2017, to replace an earlier version that was published in error. Please download this article again to view the correct version.
